# Schwann-cell-derived CMTM6 restricts radial axonal growth

**DOI:** 10.1038/s41467-020-18886-8

**Published:** 2020-10-07

**Authors:** Nimrod Elazar, Elior Peles

**Affiliations:** Department of Molecular Cell Biology, Weizmann Institute of Science, Rehovot, 76100 Israel

**Keywords:** Cellular neuroscience, Schwann cell

## Abstract

Myelinating Schwann cells control the diameter of the axons they ensheath by an unknown mechanism. In a recent article in *Nature Communications*, Eichel and colleagues identify the tetraspan protein CMTM6 as a regulator of axonal caliber.

Axons produce action potentials over their entire length with the speed of propagation proportional to the square root of axonal diameter^1^. The evolution of large and complex organisms required fast nerve conduction velocities without dramatically increasing axonal and subsequently nervous system dimensions^2^. This necessity gave rise to myelin, a lipid rich multilamellar membrane sheath produced by Schwann cells in the peripheral nervous system (PNS) that enables fast propagation of electrical signals along myelinated axons. Optimal conduction along these axons is determined by several factors, including unique characteristics of the myelin sheath^3^, molecular organization of the axolemma^4^, and the axonal diameter^5^. In addition, myelinated PNS axons display a typical reduction in the axonal cross-sectional area at nodes of Ranvier (NOR)^6^ where the high concentration of voltage-gated sodium channels enables the regeneration of the action potential. This nodal constriction lowers the threshold for action potential generation and further minimizes the internodal (i.e., below the compact myelin) caliber required to achieve a faster conduction velocity. Schwann cells can directly control axonal caliber through myelin-associated proteins such as MAG^7^, however other molecular mechanisms underlying such regulation remain largely unknown.

In a recent article published in *Nature Communications*, Eichel et al.^8^ used a biochemical approach to purify the lightweight membrane fraction associated with the axon-myelin interface from sciatic nerves, which they termed the axogliasome-enriched fraction (AEF). This approach led to the identification of ~700 proteins, including CMTM6 (CKLF Like MARVEL Transmembrane Domain Containing 6), a tetraspan protein containing two extracellular loop domains, and short N- and C-terminal tails both extending into the cytoplasm. The authors found that CMTM6 is localized to the Schwann cell adaxonal membrane facing the periaxonal space and is upregulated during myelination, suggesting that it may play an important role in the development of a functional myelin unit.

To test the function of CMTM6 in myelination, the authors specifically deleted the corresponding gene in myelinating Schwann cells. However, while ablation of CMTM6 did not result in significant myelin abnormalities, it did cause an increase in the caliber of myelinated axons. This observation suggests that glial-derived CMTM6 regulates axonal caliber by confining radial growth of myelinated axons. The mice lacking CMTM6 in Schwann cells exhibited elevated sensory nerve conduction velocity (NCV) and related behavioral abnormalities. Schwann cell ablation of CMTM6 did not affect nodal or paranodal dimensions, indicating that the observed increase in NCV strongly depends on the change in internodal axonal diameter. Eichel and colleagues also studied whether Schwann cell-derived CMTM6 plays any role in maintaining the axonal ultrastructure in mature mice. They showed that similar to developmental deletion of CMTM6, removal of CMTM6 from adult myelinating Schwann cells led to an increase in myelinated axonal diameter indicating that its presence is required to continuously restrict axonal radial growth after myelination has completed.

Previous studies had shown that the absence of myelin-associated glycoprotein (MAG), which is present at the adaxonal Schwann cell membrane, leads to reduced axonal caliber attributed to decreased neurofilament phosphorylation and spacing^7^. In contrast, ablation of CMTM6 resulted in the reverse effect, i.e., an increase in axonal caliber with no apparent difference in neurofilament density or phosphorylation. Moreover, ablating MAG together with CMTM6 in myelinating Schwann cells resulted in increased of axonal diameters, similar to the conditional knock-out of CMTM6 alone, indicating that CMTM6 regulates axonal thickness through a mechanism that is independent of MAG. Indeed, Eichel and colleagues did not observe enlargement of the nodal environ, an area in which neurofilament dynamics play a key role determining nodal morphology^9^.

The results presented in this manuscript raise a number of interesting questions, chief among them is how does CMTM6 regulate axonal caliber? CMTM6 has been mostly studied in the context of anti-tumor immunity by regulating plasma membrane expression of programmed death ligand-1 (PDL1/CD274)^10^. However, no change in either expression or localization of PDL1 was observed in conditional CMTM6 KO mice suggesting that CMTM6 regulates axonal caliber in a PDL1 independent manner. Normal G-ratios measured at multiple timepoints throughout development suggest that axonal diameters and myelin thickness in CMTM6 mutant mice increase at the same time. This emphasizes the difficulty in establishing whether the increase in axonal caliber drives the production of more myelin via neuregulin-to-erbB signaling, or rather the increase in axonal caliber is secondary to the production of thicker myelin.

The axonal submembrane organization of the actin-spectrin lattice plays a key role in determining axonal caliber^11^. Tetraspanin proteins have been shown to mediate membrane-dependent cytoskeletal reorganization^12^. Interestingly, the tetraspanin-related GPM6B glycoprotein, present in Schwann cell microvilli, was shown to regulate the morphology of the nodes of Ranvier^13^, highlighting the role these proteins have in regulating axonal morphology. Eichel and colleagues propose a model in which CMTM6 or a CMTM6-containing complex interacts in trans with a yet to be identified neuronal receptor, which may regulate the underlying submembrane actin-spectrin lattice and consequently axonal caliber. However, the finding that in mice lacking MAG, CMTM6 is strongly reduced and exhibits perinuclear localization, suggest that it may restrict axonal caliber when retained in intracellular compartments as well. While future work is needed to unveil the exact mechanisms by which CMTM6 restricts radial axonal expansion, these findings add a new layer of regulation to the complexity of the peripheral nervous system. Indeed peripheral nerves contain diverse neuronal populations which differ in their axonal diameter and level of myelination. Such versatility raises the question whether differential expression of CMTM6 in different Schwann cells affects the specific morphological features of the underlying axons? Surprisingly, Eichel et al. also detected a mild increase in the diameter of non-myelinated Remak bundle axons in CMTM6 cKO mice. The Cre-reporter mouse line used in this study to ablate CMTM6 in myelinating Schwann cells is also active in developing nerve-resident neural crest cells^14^ that should drive recombination of CMTM6 in what will become Remak cells, raising the possibility the CMTM6 expressed in non-myelinating Schwann restricts axonal caliber.

Similarly to the PNS, the central nervous system (CNS) also exhibits a wide range of myelinated axonal calibers^15^. While it does not seem that myelinating oligodendrocytes express considerable amounts of CMTM6, it may be possible that oligodendrocytes express a CMTM6 paralog which regulates CNS axonal caliber. It would be interesting to test whether ectopic expression of CMTM6 in myelinating oligodendrocytes will maintain its regulatory function on CNS axonal calibers too. Finally, neuronal damage and decrease in conduction velocity are involved in the pathophysiology of various neuropathies, and it will be of interest to study if silencing of CMTM6 can increase axonal caliber and conduction velocity in animal models of these conditions. In summary, the work of Eichel and colleagues provides an important insight into how glial cells contribute to regulate axonal caliber and subsequent function in the peripheral nervous system.
